# Polypharmacy, pain-related disability, and treatment fragmentation among U.S. adults without clinician advice to limit alcohol or tobacco use: A cross-sectional analysis of the 2022 Medical Expenditure Panel Survey

**DOI:** 10.1097/MD.0000000000048927

**Published:** 2026-05-22

**Authors:** Fares Qeadan, William A. Barbeau, Philip J. Kroth

**Affiliations:** a Parkinson School of Health Sciences and Public Health, Loyola University Chicago, Maywood, IL; b Department of Biomedical Informatics, Western Michigan University Homer Stryker M.D. School of Medicine, Kalamazoo, MI.

**Keywords:** care coordination, chronic pain, healthcare utilization, polypharmacy, treatment fragmentation

## Abstract

Pain-related disability often co-occurs with complex care patterns and high medication burden. Fragmented ambulatory care and polypharmacy may heighten adverse outcomes even among adults without clinician-identified alcohol or tobacco concerns. Using a cross-sectional analysis of the 2022 Medical Expenditure Panel Survey, we estimated the prevalence of ambulatory treatment fragmentation and polypharmacy among US adults with moderate-to-severe pain who had not received clinician advice to limit alcohol or tobacco use, and assessed their associations with emergency and inpatient utilization. The analytic sample included 1547 adults reporting moderate-to-severe pain interference with work. Treatment fragmentation was defined as seeing ≥5 unique provider specialties, and polypharmacy as filling ≥5 unique prescription medications in 2022. Outcomes were the number of emergency department (ED) visits and inpatient discharges. Survey-weighted ordinal logistic regression was used to estimate adjusted associations. High treatment fragmentation and polypharmacy occurred in 27.7% and 61.5% of adults with pain, respectively. The most common specialties were general practice (39.5%) and nursing (35.8%), and the most filled medications were atorvastatin (23.9%), metoprolol (15.5%), and gabapentin (15.4%). Each additional 5 specialties seen was associated with more ED visits (adjusted odds ratio [aOR] = 1.59; 95% confidence interval [CI], 1.15–2.21) but was not significantly associated with inpatient discharges (aOR = 1.38; 95% CI, 0.92–2.05). Each additional 5 unique medications filled was associated with more ED visits (aOR = 1.47; 95% CI, 1.24–1.74) and inpatient discharges (aOR = 1.44; 95% CI, 1.19–1.74). These findings suggest that care fragmentation and polypharmacy are common among adults with pain who have not received clinician advice to limit alcohol or tobacco use and are independently associated with greater acute-care utilization. Strengthening care coordination and medication oversight may help mitigate these risks.

## 1. Introduction

Pain and painful conditions are widespread in the United States (U.S.). Conditions such as osteoarthritis, back pain, and headaches are among the top 10 reasons people seek medical care in the U.S., and the prevalence of painful health conditions has increased from 32.9% in 1997 to 1998 to 41.0% in 2013 to 2014.^[1,2]^ In 2023, 24.3% of U.S. adults reported chronic pain, and 8.5% reported limitations in life and work activities due to chronic pain.^[3]^

Pain symptoms arise from numerous medical conditions, and, in some cases, may be a disease itself.^[4,5]^ Among people with moderate or severe pain, 95.9% have a diagnosis of a primary or secondary pain-related medical condition. Likewise, patients reporting pain or chronic pain are more likely to have multiple medical and psychiatric conditions.^[6–10]^ Based on aggregated data from the 2011 to 2019 Medical Expenditure Panel Survey (MEPS), 57.1% of the U.S. adult population has at least 1 comorbidity, compared with 73.2% of U.S. adults with chronic or surgical pain.^[11]^

Treating pain and its underlying conditions often involves multiple healthcare providers and leads to substantial healthcare costs.^[7]^ A study using 2009 to 2010 Canadian administrative health data found that patients with chronic pain accounted for 58.8% of all physician visits and 54.2% of all hospital admissions.^[9]^ Moreover, 90.2% of chronic pain patients saw a family physician, and 75.9% saw a specialist physician.^[9]^ Among U.S. pain patients, most ambulatory, emergency department (ED), and inpatient hospital care is sought for pain-related conditions, and more severe pain is associated with a greater number of ambulatory visits.^[1]^ In summary, patients with pain seek more ambulatory, emergency, and hospital care. However, the specific type of ambulatory specialty care sought, and whether ambulatory care among pain patients is fragmented, remain unknown. Care fragmentation, defined as visits spread across many different healthcare providers,^[12]^ is associated with subsequent hospitalization among patients with ambulatory care-sensitive conditions and Medicare beneficiaries.^[13,14]^ However, among pain patients, the degree of ambulatory care fragmentation and its relationship to acute-care utilization remain unknown.

Given high comorbidity and healthcare utilization, it is expected that patients with pain will use more prescription medications. Studies from 2010 and earlier have found the average number of annual prescriptions filled per patient with pain ranges from 19 to 33.^[15,16]^ The medications with the highest average annual fills per pain patient were antihypertensives (5.85 fills/yr), opioids (3.01 fills/yr), hypoglycemic agents (2.07 fills/yr), antidepressants (1.62 fills/yr), statins (1.55 fills/yr), anticonvulsants (1.36 fills/yr), and anxiolytics (1.36 fills/yr).^[15]^ More recent studies have found that over one-third of commercially insured pain patients and over half of Medicaid pain patients fill prescriptions for 2 or more pain medications, with nonsteroidal anti-inflammatory drugs and opioids the most commonly filled.^[17]^ This suggests that pain patients frequently use multiple medications to treat pain and comorbidities. However, contemporary data on pain patients’ concurrent use of multiple medications across therapeutic classes, not just the number of prescriptions filled, are lacking.

The combination of potentially high treatment fragmentation and polypharmacy among pain patients poses safety concerns. High polypharmacy increases the risk of adverse drug interactions and inappropriate prescribing, which can be difficult for providers to mitigate when patients’ care is fragmented across many ambulatory providers.^[18–20]^ Patients with pain and no documented substance use disorder might be especially at risk if fragmented care leads to opioid prescribing with inadequate monitoring for opioid misuse, addiction, and overdose. In fact, patients receiving opioid prescriptions alone or along with benzodiazepines from multiple providers are at increased risk of overdose compared with patients who have only 1 prescribing provider.^[21,22]^ Thus, it is especially important to characterize the nature of treatment fragmentation and polypharmacy in pain patients without clinician-identified substance use concerns.

The present study seeks to address these gaps by using 2022 MEPS data to characterize the types of specialty ambulatory healthcare visits used by pain patients without healthcare provider advice to reduce alcohol or tobacco use and how these visits relates to polypharmacy. Specifically, we will estimate the percentage of patients with high ambulatory treatment fragmentation, defined in this study as seeing 5 or more unique specialties during 2022, and characterize the type of specialty ambulatory care sought. We will also estimate the percentage of patients with high polypharmacy and characterize the frequency of all prescription medications used by pain patients. These statistics will provide greater specificity in characterizing the type and amount of ambulatory care and prescription medication use by pain patients than has been previously described in the literature. Last, we will examine whether fragmented ambulatory care and polypharmacy are associated with increased ED or inpatient hospital utilization as these are signals of possible progression of pain-related conditions, pain, or treatment complications. We hypothesized that greater ambulatory care fragmentation and higher polypharmacy would each be associated with more emergency department visits and inpatient discharges.

## 2. Methods

### 2.1. Setting and participants

Data are from the 2022 MEPS, which is designed by the Agency for Healthcare Research and Quality (AHRQ) to provide nationally representative estimates of healthcare use and expenditures of the U.S. civilian, noninstitutionalized population. Each year a new panel is selected from a subsample of the previous year’s National Health Interview Survey’s participants. The MEPS utilizes a complex sampling design with cluster and stratified sampling and survey weighting. We conducted a cross-sectional analysis of the 2022 MEPS full-year consolidated and event-level files; exposures and outcomes were assessed over calendar year 2022.

Our study used data from the MEPS household component (HC). Most of the data collected in the HC is queried from survey participants with computer-assisted personal interviewing technology.^[23]^ Our study also utilized data collected from the preventive self-administered questionnaires, which are paper-and-pencil questionnaires. HC data on prescribed medications, medical visits, and expenditures are also collected through the medical provider component (MPC), where AHRQ contacts survey participants’ medical providers and pharmacies to collect data on medical visits and prescribed medications. AHRQ uses the MPC to impute or edit participant HC survey responses. To reduce information bias, prescription and utilization measures were derived from MEPS event files; MEPS incorporates MPC data to edit and supplement participant-reported medical visits and prescription fills.

The inclusion and exclusion criteria were set to target adults with functional pain limitations, and no reported clinician advice to reduce alcohol or tobacco use. Specifically, participants were asked the Veterans RAND 12-Item Health Survey item, “During the past 4 weeks, how much did pain interfere with your normal work (including both work outside the home and housework)?” and included if they endorsed “moderately,” “quite a bit” or “extremely.”^[24]^ Individuals were excluded if they answered “yes” to either “In the past 12 months, has a doctor, nurse, or other health care professional advised you to cut back or stop drinking alcohol?” or “In the past 12 months, were you advised by a doctor, nurse, or other health care professional to quit smoking or quit using tobacco?” Of the 22,431 MEPS participants, 1547 met the inclusion/exclusion criteria for this study. The study size was determined by the number of MEPS respondents meeting eligibility criteria in 2022; no a priori power calculation was performed because this was a secondary analysis of a fixed sample. The Loyola University Chicago Institutional Review Board (LU# 219810) classified this study as exempt (45 Code of Federal Regulations 46.104[d][4][i]) because it involved secondary analysis of publicly available, de-identified data. This study is reported in accordance with the Strengthening the Reporting of Observational Studies in Epidemiology guideline.

### 2.2. Measures

The primary exposures of this study are the number of unique ambulatory provider specialties seen (measure of ambulatory treatment fragmentation) and the number of unique prescribed medications filled (measure of polypharmacy) in 2022. Both variables are derived from data collected in the HC and MPC. The number of unique ambulatory specialties was constructed from reported office-based and hospital outpatient department visits. For each office-based or hospital outpatient visit, participants were asked about the types of providers they spoke to (e.g., medical doctor, physician assistant, nurse practitioner, etc) and if they spoke to a medical doctor, the specialty of the doctor. For each person, the number of unique specialists seen was summed across all office-based and hospital outpatient department visits in 2022. Physician assistants and nurse practitioners were counted as possible specialists, while noting that MEPS does not collect data on the specialty of these advanced practice providers. It is important to note that operationalizing treatment fragmentation as the number of unique ambulatory provider specialties ignores whether visits are concentrated with 1 provider or diffused across many providers. There are multiple indices of treatment fragmentation such as the Bice-Boxerman index or the Usual Provider of Care index that capture visit concentration.^[25–27]^ However, MEPS data do not allow medical events to be linked to specific providers for each patient, making it impossible to calculate these indices.

The number of unique prescribed medications was constructed from records of all reported prescription fills (including refills) at any retail pharmacy. Details collected from each fill include medication name, National Drug Code, dosage, and more. Each prescribed medicine fill is also mapped to the Multum Lexicon therapeutic classification system and the Multum generic drug name of the drug used most by prescribing physicians. The number of unique prescribed medications was taken as the sum of unique Multum generic drug names filled in 2022.

The primary outcomes are the total number of ED visits and the total number of hospital discharges in 2022; both are constructed by AHRQ. Upon examination of the distributions, the number of ED visits was collapsed to an ordinal variable consisting of 0 visits, 1 visit, 2 visits, and 3 or more visits. Similarly, the number of hospital discharges was collapsed into 0 discharges, 1 discharge, and 2 or more discharges.

Other independent variables include the usual source of care patients go to “if they are sick or need advice about their health,” and the filling at least 1 prescription medication that is classified as a central nervous system (CNS) agent or a psychotherapeutic agent. Clinically relevant variables include self-rated health and the number of chronic conditions present. MEPS only assesses a select number of chronic conditions in the interview and asks participants if they have ever been diagnosed with high blood pressure, heart disease (including coronary heart disease, angina, myocardial infarction, and other unspecified heart disease), stroke, emphysema, high cholesterol, cancer, arthritis, diabetes, asthma, or if they had chronic bronchitis in the past 12 months. The number of chronic conditions was determined as the number of affirmative responses to the preceding 13 chronic conditions. Demographic variables include age, sex, race/ethnicity, marital status, highest level of educational attainment, family income as a percentage of the federal poverty line, and health insurance status.

### 2.3. Statistical analysis

Categorical data were summarized with unweighted counts and survey-weighted percentages. The Rao-Scott chi-squared test was used to test for association between categorical variables. Numerical data was summarized with the 1st quartile (*Q*1), median, 3rd quartile (*Q*3), and interquartile range (IQR). The *t-test* was used to test for associations between numerical variables and exposures. Four separate ordinal proportional odds logistic regression models were used to examine the associations of the number of drugs prescribed and number of provider specialties seen with ED and hospital utilization, with adjustment for covariates. Because polypharmacy likely mediates the relationship between treatment fragmentation and ED or hospital utilization, it was excluded from models estimating the association between treatment fragmentation and ED or hospital utilization. Since no major statistical software (i.e., Statistical Analysis System [SAS; SAS Institute Inc., Cary], R [R Foundation for Statistical Computing, Vienna, Austria], or Stata [StataCorp LLC, College Station]) currently has an implementation of formal tests of the proportional odds assumption in complex sampling design settings, we created forest plots to compare the proportional odds regression coefficients of the primary exposures with the coefficients from separate binary logistic regression models (Fig. S1, Supplemental Digital Content 1 and Fig. S2, Supplemental Digital Content 2). This graphical assessment of the proportional odds assumption is similar in spirit to the Brant test,^[28]^ and did not show violations of the proportional odds assumption. Graphical checks found age to be linear with the log odds of ED visits and nonlinear with the log-odds of hospital discharges. Thus, age was modeled linearly with the log-odds scale for ED visits and as a piecewise linear spline with a knot at 32 years for hospital discharges. All analyses were weighted and used Taylor series linearization to estimate design-based variances. All hypothesis tests are 2 sided at a 5% significance level. All analyses were conducted in SAS version 9.4 (SAS Institute, Inc.). Analyses used complete-case records for model covariates, which led to the exclusion of 78 of 1547 observations from analyses, corresponding to an unweighted missingness rate of 5%.

## 3. Results

A total of 1547 adults reported at least moderate pain interference with work and were not advised by a healthcare provider to limit alcohol or tobacco consumption. Of the 1547 participants with moderate-to-severe pain, the prevalence of high treatment fragmentation, defined as seeing 5 or more unique ambulatory specialists, was 27.7%, and the prevalence of high polypharmacy, defined as filling 5 or more unique prescription medications over the year, was 61.5% in 2022 (Table 1) indicating that a substantial proportion of U.S. adults with functionally limiting pain experience fragmented care and high medication burden, even without clinician advice to reduce alcohol or tobacco use. Most of these individuals were female (60.6%), non-Hispanic White (68.1%), and married (47.6%; Table 1). The median age was 63 years (IQR = 24). Most individuals’ highest educational attainment was a high school diploma, general educational development credential, or higher (75.2%); most had a middle or high family income (61.9%), and had health insurance (97.0%). Most individuals rated their health as good, very good, or excellent (57.7%) and the median number of comorbidities was 3 (IQR = 3; Table 1).

**Table 1:** Ambulatory treatment fragmentation, polypharmacy, and participant characteristics among U.S. adults with moderate-to-severe functional pain limitations and no clinician advice to limit alcohol or tobacco use, 2022.

	Treatment fragmentation*			Polypharmacy†			Overall
	Low	High	p	Low	High	*P*	
Treatment fragmentation,* n (%‡)						<.001	
High (≥5)	–	–		50 (8.2)	417 (39.4)		467 (27.7)
Low (<5)	–	–		422 (91.8)	624 (60.6)		1046 (72.3)
Polypharmacy,† n (%‡)			<.001				
High (≥5)	624 (52.4)	417 (88.9)		–	–		1050 (61.5)
Low (<5)	422 (47.6)	50 (11.1)		–	–		497 (38.5)
CNS or Psychotherapeutic Rx,§ n (%‡)			<.001			<.001	
Yes	705 (64.8)	390 (84.2)		214 (42.0)	890 (85.9)		1104 (69.0)
No	341 (35.2)	77 (15.8)		283 (58.0)	160 (14.1)		443 (31.0)
Usual source of care, n (%‡)			<.001			<.001	
None	143 (16.4)	33 (9.0)		105 (24.3)	82 (8.6)		187 (14.5)
Facility	155 (15.5)	48 (10.6)		83 (17.4)	124 (12.0)		207 (14.0)
Person or person in facility	725 (68.1)	386 (80.4)		292 (58.3)	837 (79.4)		1129 (71.5)
Number of comorbidities, median (*Q*1, *Q*3)	2 (1, 4)	3 (2,5)	<.001	1 (0, 2)	3 (2, 5)	<.001	3 (1, 4)
Self-rated health, n (%‡)			.004			<.001	
Excellent or very good	189 (20.7)	49 (11.0)		134 (28.0)	111 (11.8)		245 (18.0)
Good	406 (39.3)	181 (39.3)		191 (42.5)	412 (37.9)		603 (39.7)
Fair	346 (31.8)	184 (40.1)		139 (24.5)	399 (39.6)		538 (33.8)
Poor	96 (8.2)	48 (9.7)		30 (5.0)	117 (10.7)		147 (8.5)
Health insurance, n (%‡)			<.001			<.001	
Any private	426 (46.8)	225 (57.5)		238 (53.7)	424 (47.1)		662 (49.6)
Public only	587 (49.4)	241 (42.5)		226 (38.7)	622 (52.8)		848 (47.4)
Uninsured	33 (3.9)	1 (0.0)		33 (7.6)	4 (0.1)		37 (3.0)
Family income,‖ n (%‡)			<.001			.021	
High income	261 (29.9)	150 (36.0)		156 (36.2)	262 (28.7)		418 (31.6)
Middle income	248 (28.3)	135 (35.8)		128 (30.2)	262 (30.3)		390 (30.3)
Low income	201 (17.8)	76 (12.0)		87 (15.1)	195 (16.8)		282 (16.1)
Near poor	74 (4.8)	33 (4.6)		24 (2.3)	84 (6.2)		108 (4.7)
Poor/negative	262 (19.2)	73 (11.7)		102 (16.2)	247 (18.0)		349 (17.3)
Education, n (%‡)			<.001			.152	
Graduate degree	90 (7.3)	66 (15.2)		44 (8.6)	115 (10.0)		159 (9.5)
Bachelor’s degree	129 (13.6)	72 (14.7)		87 (17.5)	120 (11.9)		207 (14.1)
High school diploma or GED	523 (54.0)	219 (46.4)		214 (47.4)	541 (54.1)		755 (51.6)
No degree	174 (12.4)	42 (6.9)		87 (12.4)	136 (10.0)		223 (10.9)
Other degree	124 (12.8)	67 (16.7)		60 (14.0)	135 (13.9)		195 (14.0)
Race/ethnicity, n (%‡)			<.001			.016	
NH White	631 (62.9)	362 (81.1)		297 (63.4)	718 (71.1)		1015 (68.1)
NH Black Only	173 (14.6)	59 (10.2)		72 (14.2)	164 (12.7)		236 (13.2)
Hispanic	187 (16.1)	30 (4.7)		97 (17.0)	124 (10.2)		221 (12.8)
NH other race or multiracial	55 (6.4)	16 (4.0)		31 (5.4)	44 (6.0)		75 (5.8)
Marital status, n (%‡)			.004			<.001	
Married	430 (45.7)	210 (54.2)		230 (50.0)	421 (46.1)		651 (47.6)
Divorced or separated	250 (19.1)	116 (18.5)		111 (18.4)	264 (20.1)		375 (19.5)
Widowed	204 (15.3)	86 (16.5)		63 (7.9)	234 (20.4)		297 (15.6)
Never married	162 (19.9)	55 (10.8)		93 (23.7)	131 (13.3)		224 (17.3)
Sex, n (%‡)			.012			<.001	
Male	399 (42.2)	141 (32.4)		215 (48.1)	337 (34.0)		552 (39.4)
Female	647 (57.8)	326 (67.6)		282 (51.9)	713 (66.0)		995 (60.6)
Age, median (*Q*1, *Q*3)	62 (47, 73)	67 (58, 77)	<.001	56 (40, 67)	67 (56, 77)	<.001	63 (50, 74)
Number of ED visits, n (%‡)			.038			<.001	
0	759 (72.5)	300 (62.6)		406 (81.0)	682 (63.4)		1088 (70.2)
1	191 (18.2)	106 (22.4)		71 (14.2)	231 (22.3)		302 (19.2)
2	54 (4.9)	28 (8.8)		12 (2.7)	70 (7.9)		82 (5.9)
≥3	42 (4.4)	33 (6.1)		8 (2.1)	67 (6.5)		75 (4.8)
Number of hospital discharges, n (%‡)			.023			<.001	
0	874 (84.7)	358 (76.7)		458 (92.9)	805 (76.4)		1263 (82.7)
1	135 (12.1)	85 (18.8)		33 (5.8)	190 (18.7)		223 (13.8)
≥2	37 (3.2)	24 (4.5)		6 (1.3)	55 (4.9)		61 (3.5)

ED = emergency department, GED = general educational development, n = unweigthed frequency, NH = nonHispanic, p = Rao-Scott chi-squared test for independence for categorical data or *t* test for numeric data.

* Treatment fragmentation refers to the number of distinct ambulatory specialties seen. High treatment fragmentation = 5 or more specialties. Low treatment fragmentation = fewer than 5 specialties.

† Polypharmacy refers to the number of unique prescription medications filled. High polypharmacy = 5 or more prescription medications. Low polypharmacy = fewer than 5 prescription medications.

‡ Weighted column percent.

§ Whether particpant filled at least 1 central nevervous sytem or psychotheraptuic prescription medicine.

‖ Family income as percentage of federal poverty line.

### 3.1. Treatment fragmentation and healthcare utilization

The most common specialty seen at office-based visits or hospital outpatient department visits was general practice medical doctors (MDs) with 39.5% of individuals reporting at least 1 visit with a general practice doctor in 2022. Family practice (35.8%), ophthalmology (29.3%), orthopedics (23.1%) and other MD specialists (19.8%) were also commonly reported physician specialties. Nurse/nurse practitioners (35.8%) and physician assistants (17.4%) were also commonly seen at office-based or outpatient visits (Table 2).

**Table 2:** Frequency and percentage of office-based and hospital outpatient department specialty visits by treatment fragmentation.

Speciality	Treatment fragmentation*		Overall n (%†)	*P* ‡
	Low n (%†)	High n (%†)		
General practice	372 (34.1)	250 (53.5)	622 (39.5)	<.001
Nurse/nurse practitioner§	284 (26.5)	281 (60.0)	565 (35.8)	<.001
Family practice	290 (26.6)	223 (47.8)	513 (32.5)	<.001
Ophthalmology (eyes)	215 (19.1)	282 (56.1)	497 (29.3)	<.001
Orthopedics	166 (15.3)	189 (43.5)	355 (23.1)	<.001
Other DR specialty‖	126 (10.9)	202 (43.0)	328 (19.8)	<.001
Physician’s assistant§	106 (9.6)	163 (37.9)	269 (17.4)	<.001
Internal medicine (internist)	134 (11.6)	139 (31.4)	273 (17.1)	<.001
Cardiology (heart)	108 (9.3)	174 (34.9)	282 (16.4)	<.001
Neurology	52 (5.2)	123 (26.4)	175 (11.0)	<.001
Dermatology (skin)	49 (4.5)	112 (26.1)	161 (10.5)	<.001
Psychiatry/psychiatrist	66 (6.6)	71 (16.4)	137 (9.3)	<.001
Gastroenterology	61 (5.1)	92 (19.0)	153 (8.9)	<.001
Urology	42 (3.5)	96 (21.7)	138 (8.6)	<.001
Allergy/immunology	36 (3.9)	75 (17.0)	111 (7.6)	<.001
Gynecology/obstetrics	36 (4.5)	67 (14.6)	103 (7.3)	<.001
General surgery	39 (4.0)	76 (14.6)	115 (6.9)	<.001
Otorhinolaryngology (ear, nose, throat)	25 (2.4)	82 (16.7)	107 (6.4)	<.001
Endocrinology/metabolism (diabetes, thyroid)	37 (3.3)	50 (11.9)	87 (5.7)	<.001
Oncology (tumors, cancer)	34 (2.9)	75 (12.9)	109 (5.7)	<.001
Pulmonary	25 (2.1)	72 (14.0)	97 (5.4)	<.001
Nephrology (kidneys)	18 (2.5)	54 (11.8)	72 (5.1)	<.001
Rheumatology (arthritis)	28 (2.7)	52 (10.7)	80 (4.9)	<.001
Radiology	9 (0.5)	28 (5.9)	37 (2.0)	<.001
Osteopathy (DO)	7 (0.9)	12 (2.4)	19 (1.3)	.099
Pediatrician	3 (1.6)	2 (0.4)	5 (1.2)	.138
Anesthesiology	1 (0.0)	19 (4.2)	20 (1.2)	<.001
Hematology (blood)	8 (0.6)	9 (2.5)	17 (1.1)	.008
Physical medicine/rehab	7 (0.7)	8 (1.7)	15 (1.0)	.106
Geriatrics (elderly)	5 (0.4)	5 (0.7)	10 (0.4)	.325
Plastic surgery	2 (0.2)	4 (0.9)	6 (0.4)	.078

Unless otherwise indicated, all specalists are medical doctors.

* High treatment fragmentation is defined as seeing ≥ 5 unique specialties. Low treatment fragmentation is defined as seeing < 5 unique specialties.

† Weighted column percent. The % of people in the column that saw the indicated specialist at least once in 2022.

‡ Rao-Scott chi-squared test.

§ Non-medical doctors provider.

‖ Other medical doctors specialty provider.

Only 27.7% of individuals reported high treatment fragmentation, defined as seeing 5 or more unique specialties (Table 1). As expected, high treatment fragmentation was associated with a higher likelihood of engaging with different specialties, including primary care specialties (Table 2). High treatment fragmentation was associated with a 19.4 percentage-point absolute increase in the prevalence of reporting a visit with a general practice physician (high fragmentation: 53.5%, low fragmentation: 34.1%; *P* < .001). Similarly, treatment fragmentation was associated with an absolute increase in the prevalence of visiting family practice physicians (high: 47.8%, low: 26.6%, *P* < .001), internal medicine physicians (high: 31.4%, low: 11.6%; *P* < .001), nurse/nurse practitioners (high: 60.0%, low: 26.5%; *P* < .001) and physician assistants (high: 37.9%, low: 9.6%; *P* < .001). Individuals with high treatment fragmentation had a higher prevalence of reporting a person or a person in a facility as their usual source of healthcare (80.4%) than individuals with low treatment fragmentation (68.1%; *P* < .001; Table 1).

High treatment fragmentation co-occured with high polypharmacy, as 88.9% of individuals with high treatment fragmentation also reported high polypharmacy, compared with only 52.4% of individuals with low treatment fragmentation (*P* < .001; Table 1). High treatment fragmentation also co-occured with filling at least 1 CNS or psychotherapeutic agent (84.2% vs 64.8%; *P* < .001; Table 1). Individuals with high treatment fragmentation tended to have more comorbid chronic conditions (*P* < .001) and were more likely to be non-Hispanic White (*P* < .001), female (*P* = .012), older (*P* < .001), married (*P* = .004), to rate their health as “fair” (*P* = .004), to have private insurance (*P* < .001), and to fall in middle- or high-income brackets (*P* < .001) compared with individuals with low treatment fragmentation (Table 1).

Individuals with high treatment fragmentation had a 9.8 percentage-point higher prevalence of reporting 1 or more ED visits (*P* = .038; Table 1). After adjustment for covariates, this association persisted, as every 5 additional unique specialties seen was associated with 59% increased odds of more ED visits (adjusted odds ratio [aOR] = 1.59; 95% confidence interval [CI], 1.15–2.21; Table 3). Individuals with high treatment fragmentation had an 8.0 percentage-point higher prevalence of reporting 1 or more hospital discharges compared with people with low treatment fragmentation (*P* = .023; Table 1), but this association was nonsignificant after adjustment for covariates (aOR = 1.38; 95% CI, 0.92–2.05; Table 3).

**Table 3:** Adjusted odds ratios and 95% confidence intervals for emergency department visits and hospital discharges among people with moderate-to-severe functional pain limitations and without advice from a healthcare provider to limit alcohol or tobacco consumption.

	ED visits* aOR‡ (95% CI)	Hospital discharges† aOR‡ (95% CI)
Polypharmacy§	**1.47 (1.24, 1.74**)	**1.44 (1.19, 1.74**)
Treatment fragmentation‖	**1.59 (1.15, 2.21**)	1.38 (0.92, 2.05)
Usual source of care		
None	Ref	Ref
Facility	1.14 (0.67, 1.95)	1.44 (0.62, 3.38)
Person or person in facility	1.29 (0.85, 1.96)	1.49 (0.68, 3.23)
Sex		
Female	Ref	Ref
Male	0.99 (0.69, 1.43)	1.12 (0.69, 1.82)
Race/ethnicity		
NH White	Ref	Ref
Hispanic	**2.25 (1.40, 3.62**)	1.49 (0.82, 2.72)
NH Black	0.94 (0.57, 1.56)	1.33 (0.74, 2.39)
NH other race or nultiracial	1.45 (0.76, 2.75)	**2.22 (1.19, 4.15**)
Marital status		
Never married	Ref	Ref
Divorced or separated	1.28 (0.74, 2.22)	1.37 (0.66, 2.83)
Married	0.77 (0.46, 1.29)	1.66 (0.84, 3.31)
Widowed	1.34 (0.72, 2.49)	*2.16 (0.99, 4.74*)
Education		
No degree	Ref	Ref
High school diploma or GED	*1.66 (1.00, 2.78*)	1.49 (0.79, 2.82)
Bachelor’s degree	1.28 (0.67, 2.43)	1.32 (0.59, 2.92)
Graduate degree	0.92 (0.45, 1.87)	0.87 (0.36, 2.12)
Other degree	**2.09 (1.07, 4.08**)	*2.02 (0.94, 4.34*)
Self-rated health		
Poor	Ref	Ref
Fair	0.62 (0.36, 1.07)	**0.53 (0.29, 0.96**)
Good	0.62 (0.36, 1.07)	*0.58 (0.33, 1.02*)
Excellent or very good	0.89 (0.46, 1.72)	**0.44 (0.20, 0.98**)
Health insurance		
Any private	Ref	Ref
Public only	0.81 (0.56, 1.17)	1.14 (0.73, 1.79)
Uninsured	0.72 (0.25, 2.07)	**0.09 (0.01, 0.81**)
Family income (% poverty line)¶	0.97 (0.92, 1.03)	1.01 (0.96, 1.06)
Number of comorbidities	1.06 (0.97, 1.17)	*1.10 (0.99, 1.23*)
Age < 32#	1.00 (0.94, 1.06)	0.73 (0.35, 1.52)
Age ≥ 32#	1.00 (0.94, 1.06)	1.06 (0.98, 1.15)

Bolded aORs indicated statistical significance, *P* < .05. Italicized aORs indicate effects on the boundary of significance .05 < *P* < .10.

95% CI = 95% confidence interval, aOR = adjusted odds ratio, GED = general educational development, NH = nonHispanic.

* Proportional odds logistic regression with the outcome categories: 0 ED visits, 1 ED visit, 2 ED visit, ≥3 ED visits.

† Proportional odds logistic regression with the outcome categories: 0 hospital discharges, 1 hospital discharge, ≥2 hospital discharges.

‡ Adjusted odds ratio of moving to higher outcome category/more healthcare utilization vs a lower outcome category/less heatlchare utilization.

§ Number of unique prescription medications filled. aOR is in increments of every 5 unique prescriptions. Adjusted for all other variables.

‖ Number of distinct ambulatory specialties seen. aOR is in increments of every 5 unique specialties. Adjusted for all variables except polypharmacy.

¶ aOR is in incremennts of every 100% increase.

# Age was modeled linearly with the log odds of increasing ED visits and has the same aOR for all ages. Age was modeled with a piecewise linear spline (knot at 32 years old) with the log odds of increasing hospital discharges, and has different aORs before and after 32. All aORs for age are in increments of every 5 years.

### 3.2. Prescription medications and polypharmacy

Prescription medications indicated to treat prevalent conditions such as hyperlipidemia, hypertension, diabetes, and gastroesophageal reflux were the most commonly filled among adults with moderate-to-severe functional pain limitations and without clinician advice to reduce alcohol or tobacco consumption. The most commonly reported medication was atorvastatin, with 23.9% of individuals reporting at least 1 fill of atorvastatin. Other commonly reported prescription medications included metoprolol (15.5%), amlodipine (15.4%), metformin (15.3%), lisinopril (14.9%), and losartan (10.9%). Gabapentin, which is commonly prescribed off-label for pain treatment, was prescribed to 15.4% of individuals (Table 4).

**Table 4:** Frequency and percentage of prescription drugs.

Multum generic drug name	n	% (95% CI)
Atorvastatin	422	23.9 (21.3, 26.6)
Metoprolol	269	15.5 (13.3, 18.1)
Amlodipine	281	15.4 (13.2, 17.8)
Gabapentin	262	15.4 (12.8, 18.3)
Metformin	267	15.3 (13.1, 17.8)
Levothyroxine	261	15.1 (12.9, 17.5)
Lisinopril	252	14.9 (12.8, 17.3)
Omeprazole	240	14.6 (12.1, 17.5)
Albuterol	222	13.3 (11.3, 15.6)
Losartan	195	10.9 (9.1, 12.9)
Furosemide	179	9.7 (7.9, 11.8)
Pantoprazole	155	9.2 (7.3, 11.4)
Acetaminophen hydrocodone	161	8.9 (7.5, 10.6)
Tramadol	130	7.6 (6.1, 9.4)
Hydrochlorothiazide	147	7.3 (5.9, 9.0)
Sertraline	90	7.2 (5.4, 9.7)
Meloxicam	112	6.7 (5.3, 8.4)
Tamsulosin	101	6.4 (5.0, 8.2)
Rosuvastatin	131	6.4 (5.1, 8.0)
Cyclobenzaprine	91	6.3 (4.4, 9.0)
Prednisone	104	6.3 (4.8, 8.2)
Potassium chloride	116	5.8 (4.7, 7.2)
Aspirin	98	5.8 (4.5, 7.4)
Ondansetron	74	5.6 (4.2, 7.6)
Trazodone	101	5.6 (4.1, 7.6)
Fluticasone nasal	102	5.5 (4.0, 7.5)
Bupropion	82	5.4 (4.0, 7.4)
Ibuprofen	78	5.3 (3.9, 7.2)
Apixaban	91	5.3 (4.0, 6.8)
Duloxetine	102	5.2 (4.1, 6.7)
Hydroxyzine	66	5.1 (3.6, 7.2)
Famotidine	80	5.1 (3.7, 7.0)
Montelukast	95	5.0 (3.7, 6.6)
Amoxicillin	85	4.8 (3.7, 6.3)
Insulin glargine	94	4.8 (3.8, 6.1)
Simvastatin	85	4.6 (3.5, 6.1)
Escitalopram	73	4.6 (3.1, 6.6)
Oxycodone	91	4.4 (3.3, 5.8)
Clopidogrel	79	4.4 (3.4, 5.6)
Central nervous system agents	53	4.3 (2.8, 6.4)
Cephalexin	59	4.3 (3.0, 6.1)
Allopurinol	72	4.2 (3.2, 5.6)
Carvedilol	80	4.1 (3.3, 5.2)
Ergocalciferol	70	4.1 (3.0, 5.5)
Azithromycin	76	3.9 (3.0, 5.0)
Doxycycline	58	3.9 (2.8, 5.3)
Semaglutide	56	3.8 (2.8, 5.3)
Alprazolam	56	3.7 (2.6, 5.2)
Fluoxetine	58	3.6 (2.6, 5.1)
Empagliflozin	59	3.6 (2.7, 5.0)
Glipizide	67	3.6 (2.6, 4.8)
Amoxicillin clavulanate	50	3.4 (2.4, 4.8)
Cholecalciferol	52	3.3 (2.3, 4.8)
Diclofenac topical	57	3.2 (2.3, 4.6)
Pravastatin	63	3.1 (2.3, 4.2)
Citalopram	41	3.1 (2.0, 4.7)
Tizanidine	58	3.1 (2.3, 4.2)
Acetaminophen oxycodone	57	3.1 (2.3, 4.1)
Cetirizine	54	3.0 (1.9, 4.7)
Naproxen	53	3.0 (2.1, 4.1)
Spironolactone	53	3.0 (2.1, 4.2)
Zolpidem	55	2.9 (2.1, 4.0)
Loratadine	41	2.9 (1.9, 4.3)
Buspirone	45	2.8 (2.0, 4.1)
Pregabalin	51	2.8 (1.9, 4.0)
Venlafaxine	47	2.7 (1.9, 3.8)
Methylprednisolone	48	2.7 (1.9, 3.8)
Ferrous sulfate	43	2.7 (1.7, 4.1)
Fluticasone salmeterol	50	2.7 (1.8, 3.9)
Glimepiride	39	2.6 (1.8, 3.8)
Clonazepam	41	2.6 (1.6, 4.1)
Celecoxib	38	2.6 (1.8, 3.8)
Insulin lispro	41	2.6 (1.8, 3.6)
Meclizine	41	2.6 (1.7, 3.9)
Lorazepam	39	2.4 (1.7, 3.5)
Dulaglutide	44	2.4 (1.7, 3.5)
Amphetamine dextroamphetamine	29	2.4 (1.5, 4.0)
Triamcinolone topical	45	2.4 (1.7, 3.3)
Oxybutynin	42	2.4 (1.6, 3.6)
Isosorbide mononitrate	35	2.3 (1.6, 3.5)
Nirmatrelvir ritonavir	24	2.3 (1.4, 4.0)
Sulfamethoxazole trimethoprim	36	2.3 (1.5, 3.5)
Diclofenac	37	2.3 (1.6, 3.3)
Topiramate	30	2.3 (1.4, 3.6)
Methocarbamol	36	2.3 (1.5, 3.4)
Folic acid	39	2.3 (1.5, 3.4)
Acetaminophen	47	2.2 (1.6, 3.1)
Latanoprost ophthalmic	55	2.2 (1.6, 3.1)
Sumatriptan	31	2.2 (1.4, 3.4)
Miscellaneous agents	38	2.2 (1.4, 3.3)
Baclofen	34	2.2 (1.3, 3.6)
Chlorthalidone	30	2.2 (1.2, 4.0)
Lamotrigine	32	2.1 (1.3, 3.3)
Ezetimibe	35	2.1 (1.3, 3.2)
Warfarin	41	2.1 (1.4, 3.0)
Cyanocobalamin	32	2.0 (1.3, 3.2)
Diltiazem	36	2.0 (1.3, 3.1)
Nitrofurantoin	32	2.0 (1.3, 3.0)

n = unweighted frequency, % = weighted percent of individuals with at least 1 fill, 95% CI = Wilson 95% confidence interval.

Medications falling under a CNS or psychotherapeutic Multum therapeutic class were commonly filled in this population. Among pain patients without clinician advice to cut back or stop drinking or to quit tobacco use, 69.0% reported at least 1 fill of at least 1 medication that was a CNS or psychotherapeutic agent (Table 1). Looking more specifically at Multum sub-sub therapeutic classes, 19.3% filled selective serotonin reuptake inhibitor antidepressants, 17.8% filled gamma-aminobutyric acid analogs, 17.5% filled nonsteroidal anti-inflammatory agents, 13.9% filled skeletal muscle relaxants, 13.0% filled narcotic analgesics, 12.9% filled narcotic analgesic combinations, 10.8% filled miscellaneous anxiolytics, sedatives, and hypnotics, and 9.5% filled benzodiazepines (Table 5).

**Table 5:** Frequency and percentage of Multum central nervous system or psychotherapeutic sub-sub classes.

Multum CNS/psychotherapeutic sub-sub class	n	% (95% CI)
SSRI antidepressants	278	19.3 (16.4, 22.5)
Gamma-aminobutyric acid analogs	305	17.8 (15.1, 20.8)
Nonsteroidal antiinflammatory agents	274	17.5 (15.1, 20.2)
Skeletal muscle relaxants	213	13.9 (11.4, 16.8)
Narcotic analgesics	230	13.0 (10.9, 15.4)
Narcotic analgesic combinations	230	12.9 (11.1, 15.0)
Miscellaneous anxiolytics, sedatives and hypnotics	164	10.8 (8.8, 13.1)
Benzodiazepines	156	9.5 (7.8, 11.4)
Ssnri antidepressants	154	8.2 (6.6, 10.0)
Benzodiazepine anticonvulsants	98	5.9 (4.6, 7.6)
Salicylates	98	5.8 (4.5, 7.4)
5HT3 receptor antagonists	74	5.6 (4.2, 7.6)
Phenylpiperazine antidepressants	101	5.6 (4.1, 7.6)
Miscellaneous antidepressants	82	5.4 (4.0, 7.4)
Atypical antipsychotics	68	3.8 (2.7, 5.3)
Tricyclic antidepressants	56	3.5 (2.5, 4.8)
Anticholinergic antiemetics	47	2.9 (1.9, 4.2)
Antimigraine agents	41	2.8 (2.0, 4.1)
Miscellaneous antiemetics	45	2.6 (1.9, 3.7)
Cox-2 inhibitors	38	2.6 (1.8, 3.8)
Carbonic anhydrase inhibitor anticonvulsants	30	2.3 (1.4, 3.6)
Dopaminergic antiparkinsonism agents	40	2.2 (1.5, 3.2)
Miscellaneous analgesics	47	2.2 (1.6, 3.1)
Triazine anticonvulsants	32	2.1 (1.3, 3.3)
Tetracyclic antidepressants	23	1.3 (0.8, 2.3)
Phenothiazine antiemetics	20	1.2 (0.7, 2.0)
Pyrrolidine anticonvulsants	20	1.1 (0.6, 1.9)
Dibenzazepine anticonvulsants	11	0.7 (0.4, 1.3)
Fatty acid derivative anticonvulsants	7	0.3 (0.1, 0.8)
Anticholinergic antiparkinson agents	5	0.2 (0.1, 0.6)
Analgesic combinations	4	0.2 (0.1, 0.6)
Barbiturate anticonvulsants	4	0.2 (0.1, 0.6)

n = unweighted frequency, SSRI = selective serotonin reuptake inhibitor, % = weighted percent of individuals with at least 1 filled prescribed medicine belonging to the indicated sub-sub class, 95% CI = Wilson 95% confidence interval.

Polypharmacy was common in this population with 61.5% of individuals reporting 5 or more unique prescription medications. The prevalence of filling at least 1 CNS or psychotherapeutic medication was higher among individuals with high polypharmacy (85.9%) than among those with low polypharmacy (42.0%; *P* < .001). Individuals with high polypharmacy tend to be older (*P* < .001) and have more chronic comorbidities (*P* < .001) and were also more likely to rate their health as fair or poor (*P* < .001), have public health insurance (*P* < .001), be non-Hispanic White (*P* = .016), and be female (*P* < .001; Table 1).

High polypharmacy was associated with more healthcare utilization. Individuals with high polypharmacy were more likely to report a person or person in facility as their usual source of care (79.4%) than individuals with low polypharmacy (58.3%; *P* < .001). Individuals with high polypharmacy had a 17.7 percentage-point higher prevalence of reporting at least 1 ED visit (*P* < .001), and a 16.5 percentage-point higher prevalence of reporting at least 1 hospital discharge (*P* < .001; Table 1). These associations persisted after adjustment for covariates, as an increase of 5 unique prescription drugs was associated with 47% increased odds of more reported ED visits (aOR = 1.47; 95% CI, 1.24–1.74) and 44% increased odds of more reported hospital discharges (aOR = 1.44; 95% CI, 1.19–1.74; Table 3).

## 4. Discussion

In this cross-sectional analysis of the 2022 MEPS, we found that over a quarter of adults with moderate-to-severe functional pain limitations and without advice from a healthcare provider to limit alcohol or tobacco consumption saw 5 or more ambulatory specialists and over 60% filled 5 or more unique prescription medications. High ambulatory treatment fragmentation was associated with high polypharmacy, as 88.9% of individuals who saw 5 or more specialists also reported 5 or more unique prescription medications. After adjustment for covariates in proportional odds logistic regression models, both ambulatory treatment fragmentation and polypharmacy were associated with more ED visits. After adjustment for covariates, only polypharmacy was associated with more inpatient hospital discharges.

The results of this study align with prior literature finding higher ambulatory healthcare utilization in pain patients.^[1,9]^ Our results add to the literature by describing the types of ambulatory specialty care used by pain patients. While pain patients most frequently see primary care specialties or advanced practice providers, other specialists commonly seen include ophthalmologists, orthopedists, cardiologists, and other MD specialists. We also found that higher treatment fragmentation in pain patients is cross-sectionally associated with more ED and inpatient hospital utilization. While the temporal direction between ambulatory care fragmentation and acute-care utilization cannot be inferred from our results, cohort studies in other populations, such as Medicare beneficiaries, older adults, and diabetes patients, have found that healthcare fragmentation is associated with adverse events, such as ED and hospital utilization.^[29–32]^ Additionally, we found that the association between ambulatory care fragmentation and acute-care utilization occurs despite pain patients frequently reporting care from primary care specialties (i.e., general practice, family medicine, and internal medicine) and a usual source of healthcare. Prior studies have found that higher specialty care utilization leads to worse care coordination, likely due to increased strain on primary care providers.^[33]^ However, a study of patients in the integrated Veterans Affairs health system found no association between fragmented care and all-cause hospitalization, and found that fragmented care was protective for hospitalization for ambulatory care-sensitive conditions in patients in the 90th percentile of hospitalization risk.^[34]^ The authors hypothesized that specialized care for this high-risk population offset any potential risks that care fragmentation might pose.^[34]^ This underscores that the underlying medical complexity of patients, and the healthcare settings and systems in which they receive care, modify the relationship between ambulatory care fragmentation and acute-care utilization. Given the sizeable population of pain patients in the U.S., more research is needed in this population to understand the patient, medical, and healthcare delivery settings that hinder the real-world effectiveness of fragmented specialty healthcare.

Many prior studies point to polypharmacy, drug interactions, and inappropriate medication prescribing as contributing to the adverse effects associated with care fragmentation.^[19]^ In Medicare beneficiaries, greater care fragmentation is associated with a 4% increase in prescription medications.^[35]^ In a qualitative study, primary care providers attributed healthcare fragmentation to medication errors.^[18]^ Given our finding that almost 90% of pain patients with fragmented care are also taking 5 or more prescription medications a year, pain patients may benefit from interventions that improve care coordination and prescription medicine safety.

Medication safety issues related to care fragmentation are likely salient in pain patients, as central nervous system and psychotherapeutic agents, such as opioids and gabapentinoids, are mainstays of pharmacologic pain treatment. With regard to opioids, a systematic review found the prevalence of prescription opioid misuse ranging from 21% to 29% and the prevalence of opioid addiction ranging from 8% to 12% in chronic pain patients.^[36]^ At the same time, patients with uncontrolled pain are at the greatest risk of adverse events due to prescription opioids, as they have more than 6 times the hazard of developing opioid use disorder and almost 2 times the hazard of overdose.^[37]^ Polypharmacy and care fragmentation can exacerbate these risks. Patients prescribed opioids are at greater risk of overdose when they have opioid prescriptions from multiple providers.^[22]^ Between 17% and 27% of people who use opioids also have an overlapping benzodiazepine prescription.^[38,39]^ Concomitant use of benzodiazepines and opioids is a known risk factor for overdose and this risk is further exacerbated when these prescriptions come from more than one provider.^[21,40]^

Besides opioids, gabapentinoids may also pose medication-related safety risks, as gabapentin was among the top 10 most frequently used prescription medications in pain patients. We found that 17.8% of pain patients without clinician advice to limit alcohol or tobacco consumption used gabapentinoids in 2022, a finding similar to 2018 estimates and mirroring the rise in national dispensing trends from retail pharmacies.^[41,42]^ A retrospective chart review of over 6000 patients prescribed gabapentinoids found neuropathic pain, musculoskeletal pain, and headache to be among the top 5 indications for their prescription, but also found that 29% of gabapentinoid prescriptions had conflicting or insufficient evidence for the prescribed indication.^[43]^ Given that safety concerns of gabapentinoids include, sedation, suicidal behavior, motor vehicle accidents, misuse, and respiratory depression in in patients with chronic obstructive pulmonary disease or who take opioids, some polypharmacy-related harms might be alleviated by better aligning gabapentinoid prescribing with appropriate indications.^[44–47]^

This study has several strengths and limitations. Due to the complex sampling design of the MEPS, the estimates provided in this study are representative of the U.S. noninstitutionalized adult population with moderate-to-severe functional pain limitations without advice from a healthcare provider to limit alcohol or tobacco consumption. Additionally, recall bias is greatly mitigated due to the MPC verifying participant-reported prescription medications and medical visits with pharmacies and medical providers. With regard to limitations, this study used a cross-sectional design and did not consider the temporal order of ambulatory visits, prescription medications, ED visits, and hospital discharges. Thus, it is possible that the associations described are due in part to ED or inpatient hospital events that lead to fragmented ambulatory care. Selection bias is possible because the analytic sample excluded participants advised to cut back or stop alcohol or tobacco use, which may correlate with comorbidity burden. Misclassification is possible because clinician advice to reduce alcohol or tobacco use may be incorrectly reported by survey participants. Residual confounding, especially from underlying medical complexity, may persist despite covariate adjustment. While we adjusted for the number of chronic conditions, this count only considers the 13 chronic conditions specifically queried in MEPS. Additionally, our measurement of healthcare fragmentation only includes the number of distinct ambulatory specialties seen. Although useful, this measurement of the number of distinct specialties seen would be complemented by a measurement of whether visits for a particular patient are concentrated with 1 provider or diffused across many different providers in the same specialty. Unfortunately, the MEPS lacks necessary data to compute such measures of visit concentration, such as the Bice-Boxerman index or the Usual Provider of Care index.^[25–27]^ Because MEPS is nationally representative of the U.S. civilian, noninstitutionalized population, findings generalize to adults with moderate-to-severe pain interference who meet our exclusion criteria; results may not generalize to institutionalized populations or to individuals with active alcohol/tobacco-related clinical concerns.

This study found that ambulatory treatment fragmentation and polypharmacy correlate with more ED and inpatient hospital utilization in pain patients without advice from a healthcare provider to limit alcohol or tobacco consumption. Given the medical complexity of most pain patients, this population would benefit from interventions that improve care coordination, continuity of care, and medication safety.

## Acknowledgments

This study used data from the Medical Expenditure Panel Survey (MEPS), sponsored by the Agency for Healthcare Research and Quality (AHRQ). The views expressed in this manuscript are those of the authors and do not necessarily reflect the official position of AHRQ or the US Department of Health and Human Services.

## Author contributions

**Conceptualization:** Fares Qeadan.

**Formal analysis:** William A. Barbeau.

**Investigation:** Fares Qeadan, William A. Barbeau, Philip J. Kroth.

**Methodology:** Fares Qeadan.

**Project administration:** Fares Qeadan.

**Resources:** Fares Qeadan.

**Supervision:** Fares Qeadan.

**Validation:** Fares Qeadan.

**Visualization:** William A. Barbeau.

**Writing – original draft:** William A. Barbeau.

**Writing – review & editing:** Fares Qeadan, Philip J. Kroth.




