# Supplementary figures and images for: Polypharmacy, pain-related disability, and treatment fragmentation among U.S. adults without clinician advice to limit alcohol or tobacco use: A cross-sectional analysis of the 2022 Medical Expenditure Panel Survey
Source: Medicine (Baltimore). 2026 May 22;105(21):e48927. doi: 10.1097/MD.0000000000048927 (PMC13201002; doi:10.1097/MD.0000000000048927)

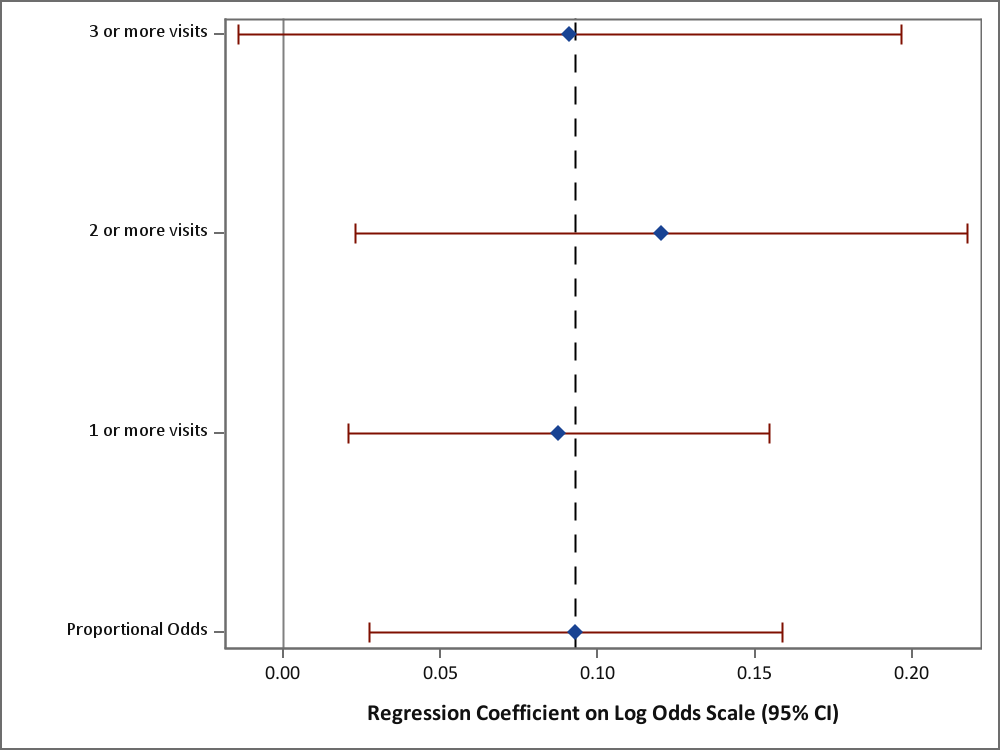

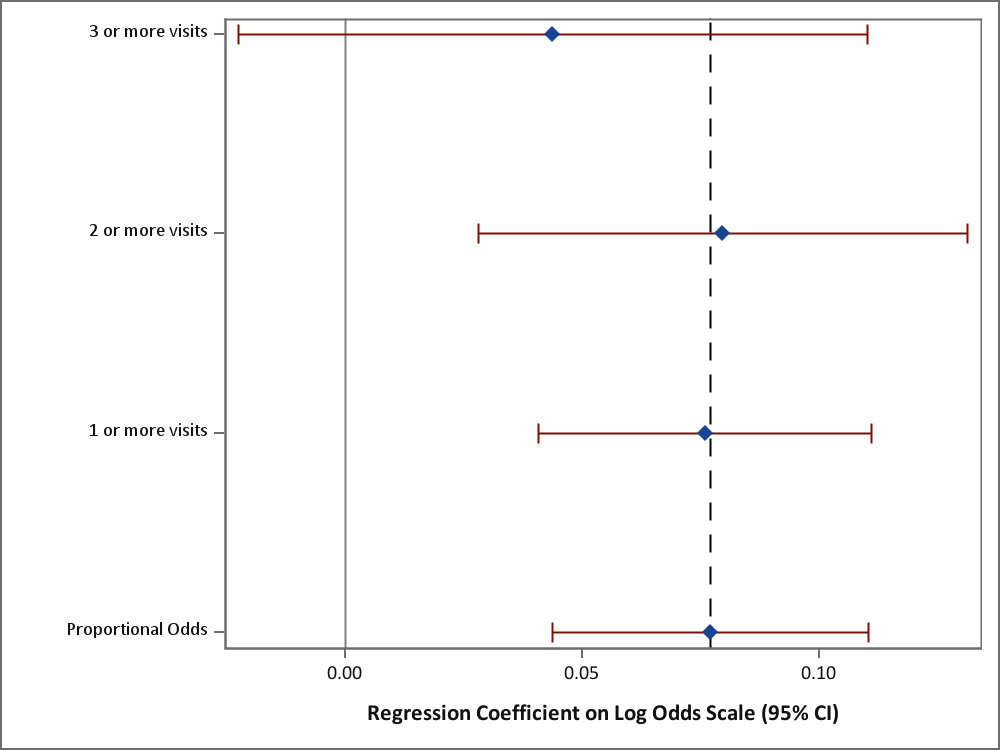


**B**

**A**

Supplement: Supplementary file 1 [file medi-105-e48927-s001.docx]

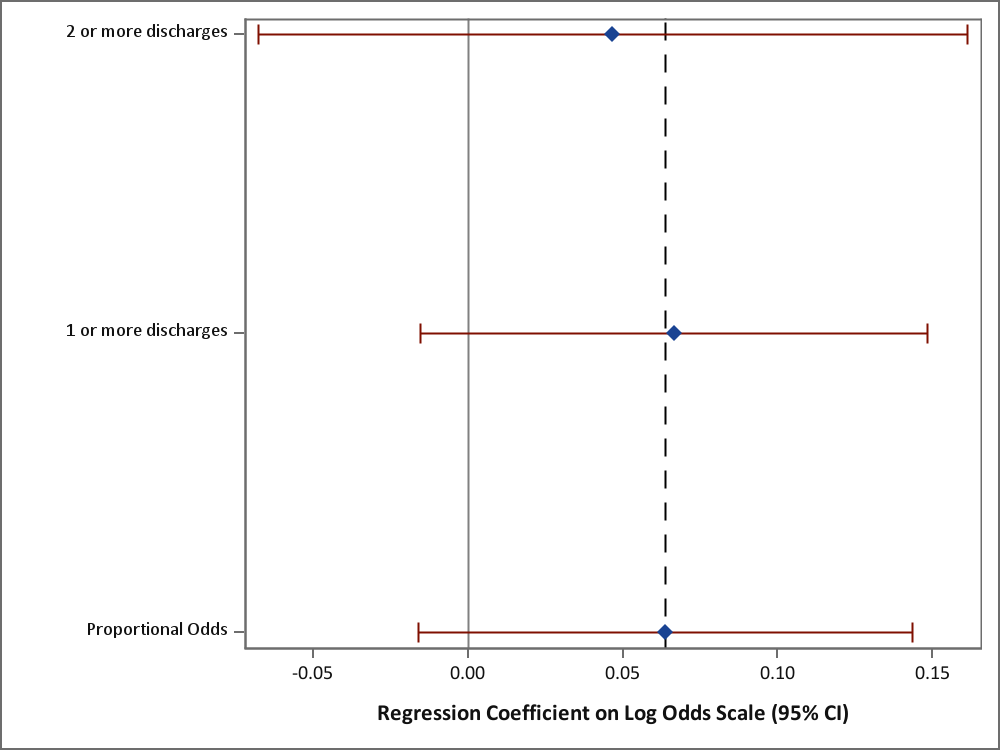

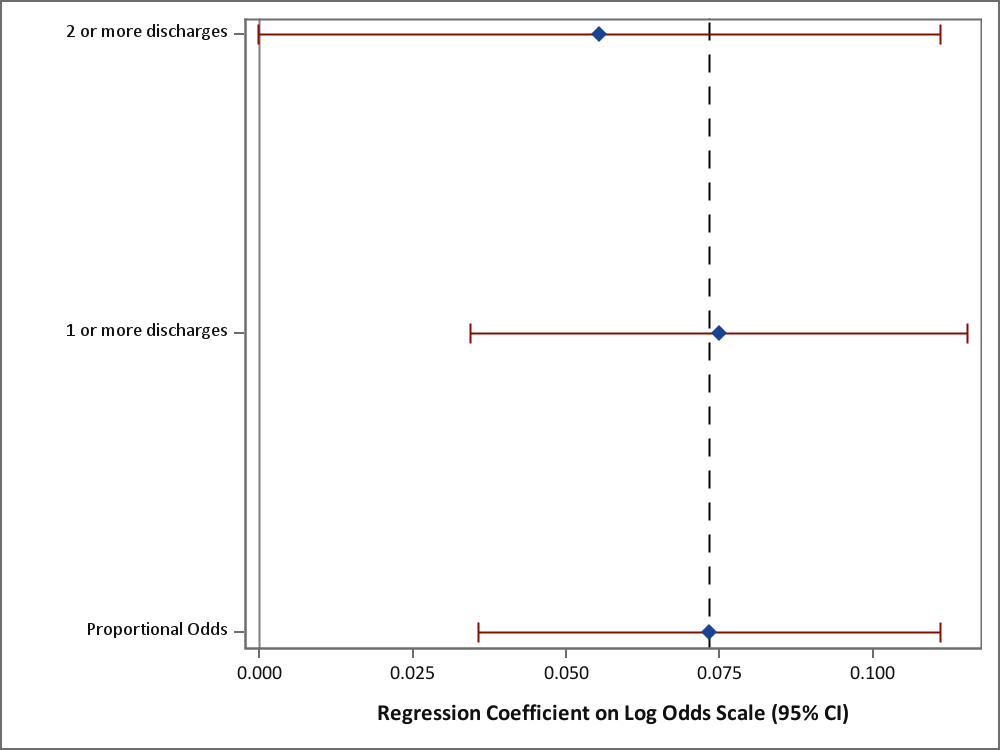


**B**

**A**

Supplement: Supplementary file 2 [file medi-105-e48927-s002.docx]
